# Putting theory into practice: a method for generating useful pre-class materials to enhance student engagement

**DOI:** 10.15694/mep.2018.0000148.1

**Published:** 2018-07-20

**Authors:** Andrew Binks, Renee LeClair

**Affiliations:** 1 Virginia Tech Carilion School of Medicine; 2 Virginia Tech Carilion School of Medicine

**Keywords:** preparation materials, integrated curriculum, student engagement, faculty development

## Abstract

This article was migrated. The article was marked as recommended.

Engaging medical students in pre-class preparation continues to be a challenge for integrated and student-centered curricular models. If assigned readings are too lengthy, complex or poorly aligned with the in-class expectations then student pre-class engagement is diminished, and consequently so is the ability of the student to engage in the active classroom. Although much has been published on the rationale and necessity of pre-class materials ( Parmelee and Michaelsen, 2010; Ewell and Rodgers, 2014; Shin and Brock, 2017), as well as the diversity of pre-class materials ( Montemayor, 2002), faculty lack a resource that outlines *how* to generate such a resource. The gap in faculty development on this front can generate curricular frustration where faculty are aware the resource they are providing isn’t ideal, student choose not to use it, and this negatively impacts the learning experience. Outlined here are 12 tips for generating pre-class materials that will promote enhanced learner preparation allowing for an elevated level of learning and reduced frustration for faculty and learners.

## Introduction

The philosophy of the flipped classroom is that ‘classwork is done at home and homework is done in class’. However, the flipped or active classroom often fails because the students fail to prepare (i.e. they don’t do the classwork at home). Poor student preparation inevitably leads to student confusion and disengagement in the active classroom and/or time costly remediation of the content by the faculty member before the ‘homework’ can be started in class ( Knewton, 2014; Bowdon, 2015). Common impediments to proper preparation include lack of appropriate study culture within a mixed pedagogy curriculum where pre-reading is of varying value to the student ( Nist and Kirby, 1989), and/or the assignment of unrealistic ( Klatt and Klatt, 2011), unappealing or inappropriate resources ( Persky and Hogg, 2017) for class preparation. Cultural barriers can be overcome by repeatedly providing clear and consistent expectations prior to class. However, these expectations will never be met unless the preparatory material is concise, focused, devoid of extraneous content, and is attractive while having minimal extrinsic load. The following are 12 tips to improve the likelihood of student preparation and consequently promote their engagement in the classroom.

## Tip 1: Recognize that integrated curriculums require changes in pre-class preparation materials

The majority of medical programs have moved away from discipline-based delivery and currently use some form of integrated curricular format ( VanTassel-Baska and Wood, 2010). Despite this shift, the traditional required textbook remains discipline-based. This misalignment can result in a single integrated class session requiring numerous different textbook resources. So while an integrated curricular model enhances many aspects of learning ( VanTassel-Baska and Wood, 2010), it makes using traditional textbooks cumbersome and disjointed for students. The problem can be exacerbated if full-chapters are assigned that include material irrelevant to the class-session. Similarly, full-chapter designations are incompatible with the overall trend to reduce traditional lecture or student contact time. These two major elements, change in curricular structure and reduced delivery time, should encouraged faculty to rethink preparation materials both in the context of the content and the need for an efficient, focused resource.

## Tip 2: Review your class session, objectives and assessment to ensure alignment

The value of the provided resource will ultimately be judged by how well it 1) addresses the session-level learning objectives, 2) supports the in-class experience and 3) assists in preparation for formative or summative assessment ( Brost and Bradley, 2006). Reduction in overall contact time has removed the luxury of redundancy/repetition between content in preparation materials content delivered in class. We suggest the role of preparation material is to provide a foundational support of basic concepts and the learning objectives associated with preparation should be aligned with verbs that are low on Bloom’s taxonomy (e.g. list, describe, explain). Resources covering basic concepts are manageable for the learner and should support the application process in the active classroom. The learning objectives for the class-session and assessment should be higher on Bloom’s taxonomy (e.g. analysis, apply and evaluate) and require integration or application of the basic concepts provided in the preparation. It is unrealistic to expect students to enter the classroom having independently achieved this higher level of learning ( Bean, 1996). Having students arrive with a foundational framework increases the likelihood a higher level of learning can be achieved through classroom-based application. By reviewing all elements of a session and assessment ensures the assigned preparation materials will be highly relevant to the learning experience and matched with the skill set of the learner, which collectively increases student engagement.

## Tip 3: Determine what knowledge is necessary for the students

As content experts in a specific disciple or clinical specialty, faculty have a wealth of knowledge that often makes it difficult to determine what content is truly necessary for a student to arrive to the classroom with. As mentioned above, the preparation resource does not need to move the learning to a high cognitive level. Pairing down the information into manageable bites gives the students a basic framework of knowledge or at the very least, a vocabulary to begin to discuss the topics at hand in the classroom. What is necessary is subjective, so be realistic with your expectations of what the learner really needs and what they are capable of achieving on their own. Tip 5 addresses suggestions on cutting down background which although may be necessary for the learner, it may have been previously addressed.

## Tip 4: Set realistic expectations of the learner’s time for preparation

Particularly in an integrated course format the students’ attention and time may be dispersed across a variety of disciplines (Tip 1). Identifying a resource for a one-hour session that will take the students 15 minutes (or less) to prepare is optimal (i.e. a 1:4 ratio of preparation to class time). As the preparation:classtime increases beyond 1:4 ratio the likelihood of a student to complete the task decreases. As the resource becomes too dense, long or extraneous, students disengage ( Persky and Hogg, 2017) and the in-class session suffers an unprepared audience. Therefore, asking students to prepare ‘everything’ can result in them preparing ‘nothing’ as they reach the point of being completely overwhelmed ( Hobson, 2004). Therefore, we suggest you offer ‘something’ to provide the essentials and maintain engagement, so that students read the resource and attend the class engaged and ready to apply basic knowledge.

## Tip 5: Use the integrated curriculum and prior student experiences to your benefit

An integrated curricular structure can be a major asset to your teaching if you take the time to review the content surrounding your session. You should determine the curricular elements that have occurred prior to your session such that you can draw on this knowledge rather than repeat it. This also greatly reduces the necessary information you may perceive a student needs (Tip 3). Familiarizing yourself with the nature and organization of your curriculum helps maximize how you spend your classroom time and avoid direct redundancy with complementary sessions. Similarly, students may have had the very basic concepts prior to starting medical school, such as the material covered by the MCAT. Also consider whether the very basic knowledge is even necessary for the content you wish to deliver; to draw on an analogy, if you wanted to tell someone how to boil an egg, it’s unnecessary to start with a description of a chicken.

## Tip 6: Think outside the textbook

After a significant amount of planning (Tips 1 - 5) it is time to identify or generate an effective preparation resource. Effective preparation can be achieved with any variety of material beyond a discipline-specific textbook and have similar outcomes ( Moravec *et al.*, 2010); so be creative. If textbooks are still your preference refine the reading to highlighted paragraphs or compiled PDF documents of focused material. Be conscious of student time restrictions (Tip 4) and what is necessary (Tip 3). It is likely that you can start by trimming reading assignments to sections of chapters or single figures. Be aware that if the assignment is too taxing (e.g. full chapters containing extraneous content) the effort by the student put into preparing will be reduced (something vs. nothing, Tip 4). Knowing your audience and level of learner will influence what types of alternative resources may be most appropriate ( Hobson, 2004). Millennial ( Roberts, Newman and Schwartzstein, 2012) and iGen ( Andone and Frydenberg, 2017) students may be more engaged with interactive preparations in the form of a short quiz ( Dobson, 2008), table, figure and/or shortened white paper resource. For some faculty these abbreviated preparation materials may not seem substantial enough, however these can include a reference to larger more comprehensive resources for learners wanting or needing more information. Self-learning modules or videos can also be appealing to students; YouTube has become a repository for robust and vetted material as well as a ‘go to’ resource for students ( Denis *et al.*, 2015). You may be able to convey the same knowledge in one of these formats and perhaps in a more constructive fashion (such as a quiz) depending on the topic. These resources can be more time consuming to generate but the product is likely to be better aligned with your specific learning objectives, class activities and assessment. It may be essential to develop an appropriate resource that, like the curriculum, is more integrated in fashion and less cumbersome for the students.

## Tip 7: Plan for change when developing resources

In addition to keeping preparation materials brief for students (Tip 4), there are a couple more reasons why a resource should be short. First, as previously eluded to, engagement will be higher for two or three short resources than one long one, e.g. students are more likely to watch three 7 minute videos than they are to embark on watching one that is 21 minutes long. Although this is a primary benefit for the students, it is also ideal for the faculty as many of our curricular structures are in flux, revision or renovation. By generating numerous short resources that cover only one or two basic concepts allows your material to be more agile. These smaller resources can be moved more readily and stay aligned with the new session’s objectives and content, whereas longer resources covering numerous concepts will likely bring content that is extraneous to a new session with different objectives. Keeping the faculty commitment in mind, as well as the students, helps with faculty engagement. If faculty feel that the commitment to self-generated resources will be useful beyond a single curricular year they may invest heavily in developing or identifying preparation materials that are flexible and easy to modify.

## Tip 8: Run a focus group

If Tips 6 and 7 seem overwhelming or if you find yourself ‘spoiled for choice’ after reviewing resource options this is a great time to run a focus group. Ask the user (student, fellow faculty, resident and/or assessment team) what format is most appropriate, best aligned, concise and user-friendly. Getting feedback on the front-end may save you a lot of time in the development and identification phase (Tip 7). It may be that students prefer a table vs. a short video for the content that you are delivering. In that example, you may be able to use a textbook figure and save yourself a significant amount of time on video development. You may also receive helpful feedback from users regarding the depth of content and its appropriateness for the audience. If you have an idea in mind, generate a draft and propose it to the group.

## Tip 9: Find support within your institution

Resource development may require you to learn a new skill set or access a new library resource. These are tasks that take time but it is likely your institution has IT support, instructional design teams and medical librarians to assist in this process. Depending on your institution, CME credit may even be available to learn this new skill! If you want to develop a short video, reach out to IT in advance to see what technology support is available in both hardware and software. You may be surprised at the wealth of resources available. If you are struggling to find an updated resource on a delivery topic, reach out to your medical librarians, they are ideal for assisting in comprehensive searches. Use the resources available to help assist in the process of development. If little or no technology support is available it is remarkable what can be done with a laptop and built-in microphone and some cheap (even free) recording software. There are free eBook generators available and these can provide a platform to compile highly specific content with specific images.

## Tip 10: Get peer-feedback on your developed or identified preparation materials

Feedback on all elements of a class session (learning objectives, preparation materials, in-class session, and assessment), should be part of your institutional culture. Much like the focus group (Tip 8), getting peer-feedback before implementation could help avoid some major pitfalls in the classroom. Asking a colleague to review your developed or identified preparation material is a useful habit to establish. First, it engages faculty across disciplines and can enhance the integrated curricular structure. It will also provide an alternative point-of-view on what is necessary (Tip 3) and realistic (Tip 4). Finally, it is likely that reviewers may be aware of additional integrated sessions that you can use to your benefit. Having these conversations also helps to understand how content delivery is supported across disciplines to identify overlap and synergy.

## Tip 11: Monitor the usage of your preparation materials

After contributing a significant effort into preparation materials, it is essential you monitor the students’ usage and satisfaction with the generated materials. This can be done through end-of-course evaluations, informal conversations and additional surveys. This can also be done observationally. Are students more engaged in the classroom activity? Are students more confident to ask questions in class? If you find students are not using the materials you have identified find out why and ask what they are using instead. It may be that although the resource is realistic and necessary (Tips 3 and 4), it isn’t well aligned (Tip 2). If you find students particularly like a specific type of preparation material you could try to use this format more often and let other faculty know of your successes. Students may also report not using your resource because your classroom activity requires no preparation (e.g. passive learning). If this is the case, rethink what is happening in class so that it isn’t redundant with the preparation materials. Don’t become discouraged if not everyone engages with your prepared resource immediately. Shifting to a culture of preparation is difficult even in the most ideal settings.

## Tip 12: Review assessment outcomes for your delivery

If students are set up to be successful with content this should be reflected in individual assessment outcomes. Tailoring pre-class preparation to a concise, aligned material may change several different formative and summative assessment outcomes. Ideally, students will perform better on a summative exam. Coming to class prepared should allow for greater levels of content application (higher level learning on Blooms Taxonomy) and this should translate into enhanced summative performance. But even if there is no change in summative assessment scores there are several other outcomes to monitor. Enhanced alignment (Tip 2) and being realistic with preparation materials (Tip 3) may translate into increased scores related to course organization or student engagement. Students may report less stress or feelings of being overwhelmed if extraneous content has been removed from the pre-class materials ( Saipanish, 2003). Ultimately, putting effort into pre-class materials may translate into an overall enhanced curricular culture as this process engages faculty across sessions (Tips 5 and 10). All of these aspects can be assessed through end-of -course evaluations or individual faculty evaluations.

## Conclusions

Faculty often overlook the utility, volume and relevance of the assigned pre-class preparation materials when planning a curricular session. Given the shift to integrated curriculum and a reduction in overall class time and increase in active learning, this element of session-level planning can no longer be overlooked and needs to be optimized for the user. Following some clear tips allows faculty to generate a resource that is used and valued by the students along with enhancing the overall curricular experience. Ultimately, if the identified resource is realistic, well aligned and necessary, students will engage with pre-class preparation allowing you to elevate the in-class experience focusing more on application exercises that are well-aligned with the student’s summative assessment.

## Take Home Messages

• Recognize that successful active classroom sessions require students to come to class with a basic foundation of knowledge and providing a concise resource with necessary content is required.
• Identify or develop concise focused resources that are well aligned to your objectives, in class activity and assessment, being realistic of the time constraints of the learner.
• Embrace current curricular changes as a time to rethink the utility of traditional textbook resources as a necessary and required part of the preparation experience.
• Continue to monitor the resource, its relevance and utility with the classroom and get feedback from peers and through evaluations.

## Notes On Contributors

Dr. Andrew Binks is an Associate Professor at Virginia Tech Carilion School of Medicine and a pulmonary physiologist. In his role as director of faculty development in the Department of Basic Science Education, he assists both clinical and basic science faculty in the development of active classroom sessions starting with the alignment of objectives, pre-class materials and assessment.

Dr. Renée LeClair is an Associate Professor at Virginia Tech Carilion School of Medicine and Chair of the Department of Basic Science Education. She has expertise in integrated curricular design and delivery. As Chair of the department, she is an advocate for the use of advanced teaching and assessment methods to establish robust, state-of-the-art teaching practices at VTCSOM.
