# Supplementary material for: Putting theory into practice: a method for generating useful pre-class materials to enhance student engagement
Source: MedEdPublish (2016). 2018 Jul 20;7:148. [Version 1] doi: 10.15694/mep.2018.0000148.1 (PMC10701846; doi:10.15694/mep.2018.0000148.1)
Supplement: Peer review history

# Reviewer report: Reviewer response for version 1

**DOI:** 10.21956/mep.19624.r29202

**Published:** 2019-07-20

**Authors:** Gary D. Rogers

**Affiliations:** 1 Griffith University

This review has been migrated. The reviewer awarded 4 stars out of 5 Thanks, Andrew and Renee, for sharing your clearly extensive experience in this area of educational practice in the form of useful and practical tips. The article was engaging and coherent. It could be strengthened, however, by being careful about not stating unreferenced opinions as fact. As an example, you assert that ‘students are more likely to watch three 7 minute videos than they are to embark on watching one that is 21 minutes long’. This is clearly your opinion born of experience but I would be very surprised if this kind of question hadn’t been formally studied somewhere in the educational psychology literature and proven (in which case you could provide the reference) or not. Otherwise, you could say something like ‘in our experience’ to emphasise that the assertion represents opinion rather than empirical fact. Another suggestion would be to take ‘tip 12’ on assessment quite a lot further. There is quite a bit in the literature about the deliberate use of low-stakes assessment in association with active learning activities (eg initial quizzes on the pre-reading prior to starting the active learning session) as a driver to engagement. Even if you are opposed to this idea, you probably need to say why. The other important aspect is that the design of all summative assessment (even end of course exams) is a critical driver for engagement in active learning. We can encourage learners to engage all we like but if our assessment tasks continue to reward memorisation over higher order learning outcomes like application, the student will stay at home with the books and cram in the last few days rather than seeing our beautifully designed active learning activities as ‘high yield’. Part of the answer to this may lie in open-book or open-internet exams, with questions carefully designed to require demonstration of higher order outcomes.

Reviewer Expertise:

NA

No decision status is available

# Reviewer report: Reviewer response for version 1

**DOI:** 10.21956/mep.19624.r29196

**Published:** 2019-02-06

**Authors:** Jacob Anderson

**Affiliations:** 1 University of Rochester Medical Center

This review has been migrated. The reviewer awarded 4 stars out of 5 This article is a great read, and well put together. Looking forward to implementing some of these ideas.

Reviewer Expertise:

NA

No decision status is available

# Reviewer report: Reviewer response for version 1

**DOI:** 10.21956/mep.19624.r29201

**Published:** 2018-08-17

**Authors:** Gary Hamlin

**Affiliations:** 1 Bond University

This review has been migrated. The reviewer awarded 4 stars out of 5 This article provides 12 thought-provoking tips that are well written, clear and relatable to implementation failure in engaging students in the flipped classroom with an emphasis on effective pre-readings. Rather than a “how to guide or method” the article is structured as a tips guide for a broad readership that includes teaching academics, program/curriculum managers, academic developers and learning and teaching managers. Throughout the tips the authors effectively apply core education concepts such as outcomes-based education and constructive alignment. Although not expressed as a specific tip there is clear instruction that flipped learning is not a shortcut for the busy educator. The article explains that effectively utilizing pre-readings in flipped learning, particularly in integrated curricula, needs a more deliberate, planned, evaluated, and evolving practice. Given the potential scope of the article the reviewer encourages the authors to consolidate some of the tips. There is repetition in the discussions of topics such as the alignment of and streamlining of materials. Consolidation would enable the authors to broaden and share their constructive thinking across other key education topics such as:1. Approaches to scaffolding the development of pre-reading materials to support transition to higher education and the spiral through the integrated medical/health curriculum with increasing complexity and sophistication whilst maintaining learner engagement, 2. Strategies for pre-readings, that engage diverse cohorts of learners, to support educators faced with increases in cohort size and diversity, 3. Developing an enabling and resilient academic culture, touched upon at the end of Tip 11. It seems that even a discrete topic like “generating useful pre-class materials for flipped learning” could be the basis for a book. The reviewer thanks the authors for a stimulating journey into the importance of the deliberate development of pre-readings to enhance the flipped classroom

Reviewer Expertise:

NA

No decision status is available

# Reviewer report: Reviewer response for version 1

**DOI:** 10.21956/mep.19624.r29198

**Published:** 2018-08-08

**Authors:** Stefanie Hautz

**Affiliations:** 1 I

This review has been migrated. The reviewer awarded 4 stars out of 5 Dear authors, thank you very much for your article which I very much enjoyed reading. It adds in a very practical way valuable information for all those preparing flipped classroom material. The article is thus of special interest for medical educators seeking answers in this topic and written in a clear and easily applicable way. Great work.

Reviewer Expertise:

NA

No decision status is available

# Reviewer report: Reviewer response for version 1

**DOI:** 10.21956/mep.19624.r29197

**Published:** 2018-07-23

**Authors:** Laurey Steinke

**Affiliations:** 1 UNMC

This review has been migrated. The reviewer awarded 5 stars out of 5 This article practices what it preaches. It is succinct, to-the-point and very useful. Have already shared it with colleagues and will continue to do so. Thank you for a useful resource.

Reviewer Expertise:

NA

No decision status is available

# Reviewer report: Reviewer response for version 1

**DOI:** 10.21956/mep.19624.r29200

**Published:** 2018-07-21

**Authors:** P Ravi Shankar

**Affiliations:** 1 American International Medical University

This review has been migrated. The reviewer awarded 5 stars out of 5 I enjoyed reading this short and interesting paper. Flipped classrooms and active learning strategies are being increasingly used in medical education and we expect students to come prepared to the sessions. The authors provide very useful comments about the preclass reading material. The importance of keeping the material short and focused has been highlighted. The authors mention about the decreasing attention span of the new generation of learners and the advantages of creating multiple short resources on a particular subject. I was encouraged to search for some free video creation software online and I will try using some of these in the future. I had previously thought about the discipline-based focus of textbooks and their utility in an integrated curriculum. Some authors are creating integrated textbooks and recently graduated students have produced some resources. Outside the developed nations, faculty resistance to active learning strategies is gradually decreasing with more and more faculty doing advanced courses in medical education. Resource constraints may also affect the use of active learning though the cost is steadily decreasing. These are welcome steps. This article will be of interest to all faculty involved in preparing advance reading materials for active learning sessions.

Reviewer Expertise:

NA

No decision status is available

# Reviewer report: Reviewer response for version 1

**DOI:** 10.21956/mep.19624.r29199

**Published:** 2018-07-20

**Authors:** Sateesh Babu Arja

**Affiliations:** 1 Avalon University School of Medicine

This review has been migrated. The reviewer awarded 5 stars out of 5 I thoroughly enjoyed reading this paper. It is very well written and easily understandable article. This is a very interesting article in the current scenario of many medical schools are shifting from the discipline-based curriculum to an integrated curriculum. With this move, redundancies are removed and the lecture hours are cut down in the curricula. In this context flipped classroom, pre-class materials, and pre-class preparation became the key factors in the medical education programs. Authors clearly detailed the factors or issues to be taken into consideration while implementing the flipped classroom. Authors also included one or two articles for each tip. This article is very useful for the faculty members who are involved in flipped classroom teaching method.

Reviewer Expertise:

NA

No decision status is available
